# Adnexal mass with extremely high levels of CA-125 and CA19-9 but normal Human Epididymis Protein 4 (HE4) and Risk of Ovarian Malignancy Algorithm (ROMA): Endometriosis or ovarian malignancy? A case report

**Published:** 2018-06

**Authors:** Sepideh Khodaverdi, Soheila Amini-Moghaddam, Fariba Almassi Nokiani, Neda Hashemi, Robabeh Mohammad Beigi

**Affiliations:** 1 *Endometriosis Research Center, Department of Obstetrics and Gynecology, Iran University of Medical Sciences (IUMS), Tehran, Iran.*; 2 *Minimally Invasive Surgery Research Center, Iran University of Medical Sciences (IUMS), Tehran, Iran.*

**Keywords:** Human epididymis protein 4 (HE4), Risk of ovarian malignancy algorithm (ROMA)

## Abstract

**Background:**

It has been shown that Carbohydrate antigen (CA) 125 and CA 19-9 tumor markers are useful for diagnosis and follow up of ovarian carcinoma.

**Case:**

In this case, we reported the high level of CA-125 and CA 19-9 with large right ovarian intact endometrioma and extensive involvement of omentum.

**Conclusion:**

Human Epididymis protein (HE4) and Risk of ovarian malignancy algorithm (ROMA) can be useful in differentiation between malignancies and benign pathologies with a good sensitivity and specificity value.

## Introduction

Tumor markers has been shown are useful for diagnosis and follow up of treatment in patients with gynecological malignancies. Carbohydrate antigen (CA) 125 that is a type of cell surface glycoprotein, is the best-known tumor marker for ovarian carcinoma as it rises in more than 80% of non-mucinous epithelial ovarian carcinomas (1). CA 19-9 is another tumor marker that rises in some ovarian masses although it commonly elevates in gastrointestinal and pancreatic malignancies (2-4). One of the most common benign diseases that are associated with the increased level of CA-125 is endometriosis (5-7) but it rarely elevates more than 100 IU/ml in endometriotic patients (8).

In cases of rupture of endometrioma, the serum concentration of CA-125 elevates as high as 10,000 IU/ml with varying amounts of ascites (1, 9) similar to those seen in ovarian malignancy, but in this case we reported the high level of CA-125 and CA 19-9 with large right ovarian intact endometrioma and extensive involvement of omentum.

## Case report

An 18 year old virgin girl was referred to clinic of gynecology in a university tertiary hospital with constant low grade lower abdominal pain from 2 weeks ago. Dysmenorrhea, gastrointestinal, or urinary tract symptoms was negative. She had regular menstrual cycles and her body mass index was 26.6. Because of abdominal obesity, we couldn't touch any masses in abdominal exam. 

Since she was virgin, vaginal exam was refused and in rectal exam a large cystic mass with mild tenderness in the right side of pelvis was palpable. Abdominopelvic ultrasonography revealed 14×10 cm complex multiseptate cystic mass containing solid components in the right ovary and a little free fluid in the cul-de-sac. Right ovary and uterus were normal. These data were confirmed in abdominopelvic spiral CT scan with and without contrast.

Complete laboratory tests including tumor markers requested. All laboratory tests were normal except Lactate dehydrogenase that was 253 (normal upper limit: 280 u/l). In tumor marker panel, alpha fetoprotein, β-HCG, carcinoemberionic antigen (CEA) were in normal range. The serum concentrations of CA-125 and cancer antigen 19-9 were 6484 IU/Ml and 1309 IU/Ml (reference range 35 IU/Ml). Human Epididymis protein 4 (HE4) was 50.7 and Risk of ovarian malignancy algorithm (ROMA) was 11%. CA-125 and CA 19-9 were measured by using appropriate chemiluminescent immunoassay kits (ROSHE Company, ELECSYS 2010 devices). 

Although HE4 and ROMA were in low risk for malignancy, because of very high levels of other tumor markers, ovarian malignancies were in the top of the differential diagnosis yet and after achieving written consent about cancer surgery, laparotomy with midline incision was done. A unilateral 14×10 cm cystic mass with very fine adhesions of cyst on the right side of corpus of uteri was detected. There was no free fluid or seeding in peritoneal cavity and cul-de-sac was not obliterated. 

Abdominal organs had normal view but the omentum was covered with many diffuse small endometriotic foci (black puckered lesions). In spite of chocolate-like content of the cyst and almost certain diagnosis of endometriosis frozen section confirmed the diagnosis. Then ovarian cystectomy was done and the biopsy from peritoneum was taken. Histological examination approved the endometriosis of omentum and endometrioma. The serum level of the CA-125 and CA19-9 decreased rapidly post operation.

**Ethical consideration**

The informed consent for reporting of the case was obtained from the patient.

## Discussion

We reported an 18 year old girl with lower abdominal pain and extremely high levels of CA-125 (6484 IU/Ml) and CA19-9 (1309 IU/Ml). The exact diagnosis was endometriosis. In the management of gynecologic patients, CA-125 and CA19-9 are used for differentiation between benign and malignant pathologies. In more than 80% of ovarian epithelial tumors, the serum concentration of CA-125 rises above 35 IU/ml (10) but it could be elevated in some physiologic states and benign conditions like menstruation and endometriosis.

CA-125 is secreted from the human endometrium and its concentration in the content of endometrioma could be high up to 1000,000 U/ml (11), although the concentration of CA-125 in patients with endometriosis rarely is more than 100 IU/ml (12). However, peritoneal mesothelial cells have more power than cancer cells in production of CA-125 (13). The important point in the interpretation of results is the magnitude of the elevation. It is reported that the serum levels of CA-125 >200 IU/ml are potentially associated with ovarian malignancies (14).

CA19-9 is other high-molecular-weight glycoproteins that increase in endometriosis (8, 10). It is shown along with advanced stages of endometriosis, the mean serum levels of the CA19-9 increases (4). The presented case had stage-3 endometriosis according to American Society for Reproductive Medicine Revised Classification of Endometriosis (16).

In this case, we had very high level of CA-125 without malignancy. The patient had diffuse endometriosis patch on the omentum like the cases reported by Agha Hosseini et al (15). The common things between these two cases were unruptured ovarian endometrioma and black puckered lesions of endometriosis of the omentum. Our assumption is that involvement of omentum by endometriosis is the cause of such an exaggerated increase in the serum level of CA-125 and CA19-9 without ovarian malignancy. Especially since the omentum has a very extensive surface, consequently there is very large amount of ectopic endometrium for secreting these tumor markers.

In the literature every high levels of serum CA-125and/or CA19-9 in endometriosis as high as 9537 IU/ml and 15653 IU/ml are reported in a patient with ruptured ovarian endometrioma (1, 8, 17) but in our patient we had extraordinary levels of CA 125 and CA 19-9: 6484 IU/Ml and 1309 IU/Ml with enraptured endometrioma.

Other points that were observed in this case were the normality of the HE4 and ROMA despite every high level of CA-125 and CA19-9. Sensitivity of HE4 and CA-125 for discriminating ovarian cancer from benign pathologies was 86.2% and 93.1% for ROMA and the specificity was 87.4, 78.9 and 90.7 % for HE4, Ca-125 and ROMA (18). Since CA-125 is a nonspecific tumor marker, it is shown that determination of HE4 while there is a high level of CA-125 levels could confirm the benign nature of ovarian endometrioma (19, 20).

## Conclusion

In conclusion, we may be faced with very high levels of CA-125 and CA19-9 in many advanced stages of endometriosis even without leakage or rupture of endometrioma. This happens particularly when the gastrointestinal organs or omentum is involved. As it has been shown in Ortiz-Muñoz *et al* study, in this situation HE4 and ROMA could be useful in differentiation between malignancies and benign pathologies with a good sensitivity and specificity value (18).

## Conflict of interests

All the authors declare no conflict of interest for this project.
